# Self-Regulatory Capacities Are Depleted in a Domain-Specific Manner

**DOI:** 10.3389/fnsys.2017.00070

**Published:** 2017-09-28

**Authors:** Rui Zhang, Ann-Kathrin Stock, Anneka Rzepus, Christian Beste

**Affiliations:** ^1^Cognitive Neurophysiology, Department of Child and Adolescent Psychiatry, Faculty of Medicine of the TU Dresden, Dresden, Germany; ^2^Experimental Neurobiology, National Institute of Mental Health, Klecany, Czechia

**Keywords:** ego depletion, backward inhibition, EEG, neurophysiology, task switching

## Abstract

Performing an act of self-regulation such as making decisions has been suggested to deplete a common limited resource, which impairs all subsequent self-regulatory actions (ego depletion theory). It has however remained unclear whether self-referred decisions truly impair behavioral control even in seemingly unrelated cognitive domains, and which neurophysiological mechanisms are affected by these potential depletion effects. In the current study, we therefore used an inter-individual design to compare two kinds of depletion, namely a self-referred choice-based depletion and a categorization-based switching depletion, to a non-depleted control group. We used a backward inhibition (BI) paradigm to assess the effects of depletion on task switching and associated inhibition processes. It was combined with EEG and source localization techniques to assess both behavioral and neurophysiological depletion effects. The results challenge the ego depletion theory in its current form: Opposing the theory’s prediction of a general limited resource, which should have yielded comparable effects in both depletion groups, or maybe even a larger depletion in the self-referred choice group, there were stronger performance impairments following a task domain-specific depletion (i.e., the switching-based depletion) than following a depletion based on self-referred choices. This suggests at least partly separate and independent resources for various cognitive control processes rather than just one joint resource for all self-regulation activities. The implications are crucial to consider for people making frequent far-reaching decisions e.g., in law or economy.

## Introduction

Self-regulation (also known as willpower) can be broadly defined as exerting cognitive control to change our responses and is a key to successes (Hofmann et al., 2012; Baumeister, 2014). In daily life, we however often fail in self-regulation under some typical circumstances, for instance after a long workday. The self-regulatory strength model (in the following: ego depletion theory) suggests that all important activities demanding self-regulation such as overriding impulses, regulating emotions and performance, or making difficult choices and decisions, seem to draw on a common limited internal resource (Baumeister et al., 1998). It has been suggested that performing an act of self-regulation depletes some common resource and therefore impairs performance on subsequent, seemingly unrelated acts that also demand self-regulation. This reduced mental capacity or willingness to engage in volitional actions caused by prior exertion of self-regulation is referred to as “ego depletion” (Baumeister et al., 1998; Baumeister, 2003, 2014).

Making decisions can be depleting. Especially plenty of choice-making has been claimed to consume the self-regulatory resource and impair subsequent self-regulation even on tasks unrelated to decision-making (Inzlicht and Schmeichel, 2012). This depleted state, which is induced by repeatedly exerting self-related decisions, is called decision fatigue (Baumeister et al., 1998; Vohs et al., 2008). However, the findings on decision fatigue are not consistent as yet. One of the reasons for this is that some of the studies have been conducted in the field of consumer research and thus put a focus on whether or not to purchase and/or consume certain products (e.g., Dewitte et al., 2009), which does not necessarily allow for precise predictions about cognitive domains. Also, several of the studies claiming to find an ego depletion effect have actually depleted and probed their study’s participants in the same cognitive or behavioral domain (e.g., Bruyneel et al., 2006; Dewitte et al., 2009). Furthermore, Tuk et al. (2015) have suggested that depletion effects may not only depend on timing (i.e., be more consistently found in sequential as compared to simultaneous self-control requirements), but also differ between self-control requirements based on working memory vs. those based on inhibition. While such data does undoubtedly demonstrate some kind of domain-specific depletion, it does often not allow for the claim that ego depletion is truly domain-unspecific (i.e., that making self-referred choices does indeed impair other cognitive control functions that do not require the same kind of self-referred choices). Lastly, not all studies found a robust ego depletion effect; some even demonstrated opposing effects/improvements of self-control after having exerted free-willed autonomous choice (Moller et al., 2006; Converse and DeShon, 2009), or when simultaneously performing several tasks requiring inhibition-type self control (Tuk et al., 2015). Yet, others have suggested that decision fatigue may be a placebo-like effect (i.e., dependent on whether or not one believes to become exhausted by making frequent decisions, see Job et al., 2010).

Against this background, we set out to investigate whether decision-making impairs diverse, subsequent self-regulation activities even in unrelated cognitive control domains (choice-specific depletion) or if depletion is actually domain-specific (i.e., only found in the cognitive domains which have previously been strained). In the current study, we therefore compared three groups. A non-depleted control group performed a backward inhibition (BI) paradigm without being previously depleted in any way. This paradigm was chosen because it allows to assess inhibitory control, which should be strongly affected by ego depletion as it is the core to self-regulation (Hofmann et al., 2012; Diamond, 2013; Baumeister, 2014)^1^ and presumably consumes a limited internal resource (Baumeister et al., 1998, 2006; Baumeister, 2002). BI is defined as the process that inhibits the most recently performed task upon switching to a new one. It contributes to flexible task switching by suppressing the interference arising from previous tasks (Allport et al., 1994; Allport and Wylie, 1999; Mayr and Keele, 2000; Costa and Friedrich, 2012). Based thereon, we assessed the effects of self-regulatory resource depletion and domain-specific depletion by analyzing the BI effect (for detailed information see the “Materials and Methods” section) in two different depletion groups. One group performed a choice-based depleting task (choice-depletion group), while another group performed a no-choice switching task which asked the participants to categorize stimuli (switching depletion group). If general depletion, but not ego depletion, played a major role in performance modulation, we would expect worse performances following both depleting tasks as compared to the non-depletion control group. If (ego) depletion effects were choice-specific, the participants’ performance should be worse after completing the choice-depletion task than after completing the categorization-based switching depletion task. If, however, depletion was mostly domain-specific, the switching task should yield the largest impairment as it strains the same cognitive domain as the BI paradigm. As (ego) depletion effects can be moderated by depleting task duration, inter-task interim period, motivational incentives and beliefs about the availability of willpower (Hagger et al., 2010; Job et al., 2010; Hagger et al., 2016), the choice-based depletion task and the switching-based depletion task consisted of the same number of trials using the same stimuli. Also, we conducted the dependent task (the BI paradigm) directly after the depleting tasks (the first task) to minimize the inter-task interim duration. We gave the same instructions to all participants to rule out differences in motivation and beliefs about willpower in all three groups.

By now, studies of ego depletion have mainly been focusing on behavioral performance, but the underlying neurophysiological mechanisms of ego depletion have remained unclear. In contrast to this, the neurophysiological mechanisms of the related concept of “mental fatigue” are well-investigated. Experimental studies demonstrate that mental fatigue impairs attentional selection (Lorist et al., 2000, 2009; Faber et al., 2012; Hopstaken et al., 2016), which can be reflected by the visual N1. The N1 has been known to be modulated by attentional selection processes such as focusing on task-relevant stimuli (Luck et al., 1997; Hillyard and Anllo-Vento, 1998; Herrmann and Knight, 2001; Beste et al., 2010b). Mental fatigue also strongly affects response selection and conflict monitoring processes reflected by changes in the N2 amplitude (van Veen and Carter, 2002; Boksem et al., 2006; Folstein and Van Petten, 2008; Beste et al., 2015, 2017). The P3 component has often been reported to be linked to processes of context-updating and stimulus-response re-mapping (Verleger et al., 2005; Polich, 2007; Wolff et al., 2017). A decrease of the P3 amplitude was found in subjects suffering from mental fatigue, thus indicating that information evaluation processes can be influenced by mental fatigue (Boksem et al., 2006; Polich, 2007; Möckel et al., 2015). Notably, although mental fatigue shares some commonalities with ego depletion, they are not the same (Vohs et al., 2008). Mental fatigue is induced by very long performances of usually several hours (Lorist et al., 2005) and affects a broad range of processes (Parasuraman, 1979). By contrast, ego depletion is induced by manipulations of a much shorter duration. Hence, it is unclear whether the same neurophysiological processes underlie ego depletion and mental fatigue. In the current study, we therefore combined EEG (event-related potentials, ERPs) with source localization techniques (i.e., sLORETA) to answer the questions which neurophysiological processes within the processing cascade from early attentional processes to response selection mechanisms are affected by general depletion/choice-specific depletion/domain-specific depletion, how these kinds of depletion modulate the BI effect, and what functional neuroanatomical networks are involved.

## Materials and Methods

### Participants

*N* = 75 healthy subjects between 18 and 30 years of age took part in the experiment. Participants were randomly assigned to a choice group (mean age of 24.9 ± 3.1; *N* = 25; 14 females), a switching group (mean age of 23.8 ± 3.7; *N* = 25; 16 females), and a control group (mean age of 23.5 ± 3.9; *N* = 25; 19 females): While the first two groups underwent a depletion procedure (described in the following text section), the control group was not depleted, which means that they directly started their appointments with the experimental paradigm. As we used an inter-individual design, each participant only had one group and performed the experimental BI paradigm only once. Assuming small effect sizes (*f* = 0.23) and an alpha error of 0.05, this sample size yields a power of 0.95 given our experimental design (see “Statistics” section). One participant in the choice group was excluded because his responses in the depleting task demonstrated either a lack of stable personal preferences or a lack of task commitment, as indicated by an extremely low consistency rate (61%; see “Manipulation Check” section). All participants had normal or corrected-to-normal vision and no history of neurological or psychiatric disorders. Written informed consent was obtained from all participants at the beginning of the experiment. The study was approved by the institutional review board of the Medical faculty of the TU Dresden in Germany and conducted in accordance with the recommendations of the Declaration of Helsinki. All participants received a reimbursement of 20 €.

### Depleting Tasks

During the experiment, participants were seated in front of a 17 inch CRT computer monitor with a viewing distance of 57 cm. For the two kinds of depletion procedures, which were administered to the choice and switching groups only, a fixation cross and two picture stimuli left and right of it (each 10 cm high and 15 cm wide) were presented in the center of the screen. There were two trial categories (food and landscapes) and for each trial, there were two kinds of stimulus pictures (sweets and beverages/beaches and mountains). In each trial, the presented stimuli were always from different stimulus categories, but from the same trial category (i.e., sweets vs. beverages or beaches vs. mountains). Each possible stimulus combination was presented equally often and each stimulus was presented equally often on both sides. There were two experimental groups. Participants in the choice group were asked to decide which of the presented foods they would prefer to consume or which place they would prefer to visit. This means that the choice group performed the same task throughout all depletion trials, irrespective of the trial category. In contrast, participants in the categorization-based switching group were asked to indicate on which side of the fixation cross the sweets or the mountains were presented. As the categories randomly alternated throughout the depletion procedure, this resulted in the noteworthy difference that the switching group had to frequently switch between the attended task rules (i.e., categorizing foods vs. categorizing landscapes) whereas the choice group did not perform task switching (they always had to follow one rule, i.e., indicate preference). Participants responded by pressing two buttons (left and right Ctrl buttons) on a regular computer keyboard using their left and right index fingers, respectively. The stimuli remained on the screen until the participants responded. If participants did not respond within 2000 ms, a speed-up sign (German Word “Schneller!”, translating to “Faster!”) appeared above the stimuli asking participants to respond more quickly. In case of a speed-up sign, any given trial would be repeated until a response was given within less than 2000 ms. Between trials, there was a fixed 1000 ms inter-trial interval (ITI) during which a fixation cross was centrally presented.

Each depleting task (i.e., choice or switching) consisted of 800 trials divided into four equally sized blocks and took about 20 min to complete. Between all blocks, there was a fixed pause of 7 s to keep the participants from taking a rest. After two blocks, the participants received feedback about their consistency rate or accuracy in the preceding two blocks, depending on whether they were in the choice group or the categorization-based switching group. The consistency rate in the choice group was calculated to check whether the participants chose the same stimulus in all presented repetitions of a given stimulus pairing. The participants in the control group did not perform any depleting tasks and directly started with the ego depletion assessment described below so that general depletion effects (i.e., irrespective of the kind of depletion task) could be investigated by comparing the two depletion groups to the control group.

### Manipulation Check

To make sure that all participants executed the depleting tasks attentively and with sufficient commitment, we examined their performance in the depleting tasks as this can potentially affect the depletion effect. To prevent this, we excluded participants whose consistency rate (in the choice group) or accuracy (in the categorization-based switching group) was lower than 80% (mean consistency: 92.82% ± 1.1; mean accuracy: 97.36% ± 0.37).

### Assessment of Ego Depletion

To assess potential ego depletion effects, we used a modified version of the BI paradigm proposed by Koch et al. (2004), which has been used in two previous neurophysiological studies investigating different research questions (Zhang et al., 2016a,b). This paradigm covers aspects of all prominent executive functions as proposed by Miyake et al. (2000). Of note, it allows to examine inhibitory processes, which are central to the self-regulation and should thus be suitable to detect effects of self-regulatory depletion (i.e., behavioral deficits caused by the choice-based depletion procedure). At the same time, it also assesses cognitive flexibility, which requires aspects of both inhibition and working memory and is usually operationalized with task switching paradigms (Diamond, 2013). Based thereon, the BI paradigm is also suitable to reflect domain-specific (i.e., task switching-specific) depletion effects as potentially caused by the switching-based depletion procedure.

**Figure 1:**
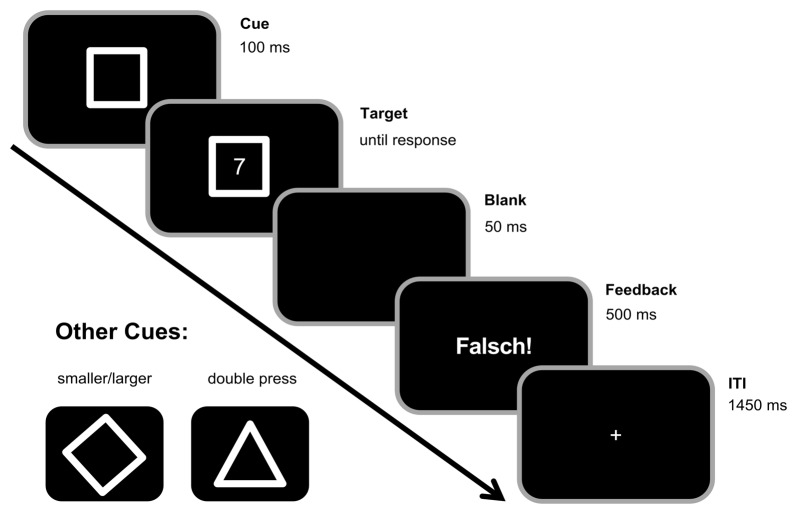
Experimental paradigm. Each trial began with the presentation of a cue in the center of the screen. A square cue indicated the odd/even task (left button press for odd numbers, right button press for even numbers). A diamond cue (see bottom left) indicated the smaller/larger rule (left for smaller than five, right for larger than five). A triangle cue (see bottom left) indicated the double press rule (simultaneous button press within the first 1000 ms after target onset). After 100 ms, the target stimulus (any digit from 1 to 9, except 5) was presented within the cue stimulus until a response was made. In the double-press task, a speedup sign (“Schneller!”, translating to “Faster!”) appeared above the cue frame in case no response was given within the 1000 ms after target onset. During the inter-trial interval (ITI) of 1500 ms, there was a 500 ms feedback for incorrect trials (“Falsch!”, translating to “Wrong!”), but no feedback/only a fixation cross in correct trials.

A square, diamond, or triangle frame were used as cues indicating task A (odd/even), task B (smaller/larger), or task D (double-press), respectively (see Figure 1). Target stimuli consisted of digits 1–9 except for 5. Each trial started with the presentation of one of the cues. After a stimulus onset asynchrony (SOA) of 100 ms, a target appeared within the cue frame. Both stayed on the screen until the participants responded by pressing one of the two Ctrl-buttons on a regular keyboard. In the odd/even task, participants should indicate whether the target digit was odd by pressing the left Ctrl-button with their left index finger or even by pressing the right Ctrl-button with their right index finger. In the smaller/larger task, they should indicate whether the target was smaller or larger than five by pressing the left Ctrl-button with their left index finger as the “smaller” response and by pressing the right Ctrl-button with their right index finger as the “larger” response. In contrast to that, participants should press both buttons simultaneously (i.e., with an asynchrony of less than 50 ms) upon target presentation in the double-press task. If participants did not respond within 1000 ms after target onset in the double-press task, a speed-up sign (German Word “Schneller!”, translating to “Faster!”) appeared above the cue asking participants to respond more quickly. Between trials, there was a fixed 1500 ms response-stimulus interval (RSI), during which a fixation cross was centrally presented. In case of a slow (more than 1000 ms in the task D, or more than 2500 ms in tasks A and B) and/or erroneous response, the German feedback “zu langsam!” (translating to “too late!”) and/or “falsch!” (translating to “wrong!”) were presented in the center of the screen during the first 500 ms of the RSI (as shown in Figure 1). Incorrect key presses, too slow responses, and non-simultaneous key-presses in the double-press task were counted as errors.

The experimental paradigm consisted of 768 trials divided into eight equally sized blocks. Each cue and target as well as each possible combination of them were randomized and occurred with the same frequency. However, neither cues nor target could be the same in two consecutive trials. Furthermore, the target in the current trial was always different from the target used in the last trial with the same cue. Within each block, each trial (except for the first two trials of each block, of course) built a triplet with the last two preceding trials. Hence, there were 752 triplets in total. All 12 possible triplet combinations (ABA; ADA; BAB; BDB; DAD; DBD; DBA; BDA; DAB; ADB; BAD; ABD) were equally frequent (±1 triplet for two of the triplet conditions in each block). Triplets with an n-2 cue repetition were categorized as BI triplets while triplets without that n-2 cue repetition were categorized as baseline triplets (compare Zhang et al., 2016b). As behavioral measures, accuracy and RTs were separately collected for each experimental condition. In this context, please note that only triplets with correct responses in all three trials were included in the analyses, thus increasing error rates. The chance level would therefore be at 12.5% when assuming a 50% chance level for each individual trial of the analyzed triplets.

A stronger BI is thought to relate to a better task switching performance as it facilitates the activation of a new task set (Mayr and Keele, 2000). However, a strong BI can also be disadvantageous, since the inhibition of a currently irrelevant task can persist over time, making it difficult to perform a previously inhibited task when it becomes relevant again (Allport et al., 1994; Allport and Wylie, 1999). Based thereon, the BI paradigm was developed to measure task set inhibition in a task switching context. In this paradigm, the BI effect is measured by assessing the time cost of overcoming the inhibition of recently abandoned task set that is relevant again (Mayr and Keele, 2000). Performance costs related to BI are observed in task sequences in which a task A or B is repeated from n-2 trials (e.g., ABA or BAB task triplet/BI triplet/BI condition), compared to when that task A or B has no n-2 trial sequence history (e.g., DBA or DAB task triplet/baseline triplet/baseline condition). According to the study of Koch et al. (2004), BI effects only appear when previous (n-1) trials require choice Go responses. Furthermore, the BI effect interacts with the preparation time when the last trials require double-press responses. To examine the BI without these distorting effects and as BI effect is operationalized via differences in the last trial of a given triplet, we only analyzed the last trial of the triplets with choice Go responses in the previous (n-1) as well as the last trial. As a consequence, BI triplets were ABA and BAB while baseline triplets were DBA and DAB. To check for potential task differences, we compared the BI effect (both for reaction time (RT) and accuracy) calculated from the ABA and DBA triplets to that from the BAB and DAB triplets. No differences in the magnitude of the BI effect in these two triplet pairs were found (all *F* < 0.57; all *p* > 0.453). We therefore averaged the two BI triplets (i.e., ABA and BAB) and the two baseline triplets (i.e., DBA and DAB) to obtain a measure for the BI condition and the baseline (BASE) condition.

### EEG Recording and Analysis

The EEG was recorded from 60 Ag–AgCl electrodes at equidistant positions with a sampling rate of 500 Hz. The reference electrode was located at Fpz and the ground electrode was located at *θ* = 58, ϕ = 78. Electrode impedances were kept below 5 kΩ. During off-line data processing, the recorded data was down-sampled to 256 Hz and a band-pass filter from 0.5 Hz to 20 Hz with a slope of 48 db/oct each was applied. A raw data inspection was conducted to remove technical artifacts, while periodically occurring artifacts such as pulse artifacts, horizontal and vertical eye movements were subsequently detected and corrected for using an independent component analysis (ICA; infomax algorithm). Afterwards, cue-locked segments were formed for trials with correct responses for all conditions separately. Segments started 300 ms prior to the locking point (cue onset) and ended 1200 ms thereafter. Next, an automated artifact rejection procedure was applied using a value difference above 200 μV in a 200 ms interval as well as an activity below 0.5 μV in a 100 ms period as rejection criteria. After that, a current source density (CSD) transformation was applied. This transformation helps to find the (electrode) location of strongest effects, which is independent of the reference (Nunez and Pilgreen, 1991). Aside from eliminating the reference potential, the CSD transformation is known to serve as a spatial filter (Nunez and Pilgreen, 1991). Such spatial filters attenuate possible effects of volume conduction (Cohen, 2014). A baseline correction was then set to a time interval from −300 ms to 0 ms before the segments were separately averaged for each condition. After that, electrodes P7, P8, P9, P10, Cz, PO1 and PO2 were selected on the basis of the scalp topography of the different ERP components. All ERP components were quantified by extracting the mean amplitude of the respective time interval. The P1, N1 and P2 ERPs were quantified at electrodes P7 and P8 following the cue (P1: 85–90 ms; N1: 140–160 ms) and following the target stimulus, which was presented 100 ms thereafter (P1: 255–275 ms; N1: 315–335 ms; P2: 400–420 ms for the control group and 425–445 ms for the choice and the switching groups). The N1 on the target stimulus was also quantified at electrodes P9 and P10 using the same time interval as for electrodes P7 and P8. At electrode Cz, the cue- and target-elicited N2 ERPs were quantified by extracting the mean amplitude of the time interval from 245 ms to 265 ms and from 400 ms to 420 ms, respectively. At electrodes PO1 and PO2, the cue and target-elicited P3 ERPs were quantified by using the time interval from 310 ms to 325 ms and from 570 ms to 595 ms, respectively. All ERP components were quantified relative to the baseline. The choice of electrodes was statistically validated using the method used by Mückschel et al. (2014). This procedure revealed the same electrodes as identified by visual inspection.

To identify functional neuroanatomical structures involved in the different depletion effects, we used sLORETA (standardized low resolution brain electromagnetic tomography; Pascual-Marqui, 2002). sLORETA reveals high convergence with fMRI data and neuronavigated EEG/TMS studies, which underlines the validity of the sources estimated using sLORETA (Sekihara et al., 2005; Dippel and Beste, 2015). sLORETA gives a single linear solution to the inverse problem, based on extra-cranial measurements without a localization bias (Pascual-Marqui, 2002; Sekihara et al., 2005). For sLORETA, the intracerebral volume is partitioned into 6239 voxels at 5 mm spatial resolution. The standardized current density at each voxel is calculated in a realistic head model (Fuchs et al., 2002) using the MNI152 template (Mazziotta et al., 2001). In this study, the voxel-based sLORETA images were compared across experimental conditions using the sLORETA-built-in voxel-wise randomization tests with 2000 permutations, based on statistical nonparametric mapping (SnPM). Voxels with significant differences (*p* < 0.01, corrected for multiple comparisons) between contrasted experimental conditions were located in the MNI-brain.

### Statistics

For tasks A and B, we excluded trials with RTs higher than 2500 ms or lower than 100 ms and for task D, we discarded trials with RTs higher than 1000 ms and lower than 100 ms (0.68% ± 0.02 of all trials). This cutoff decision was based on the article by Koch et al. (2004) and the fact that responses in tasks A and B are typically slower than those in task D. We furthermore excluded the first two trials of each block, all erroneous trials and the two trials following an error or a trial where the response was too slow. Given the exclusion criteria (i.e., requiring three consecutive correct trials for RT analysis, which lowers the chance level of correct responding to 12.5%), the mean number of the remaining trials for each triplet that entered the RT analysis was: ABA: 38.2 ± 1.6; DBA: 43.4 ± 1.4; BAB: 38.7 ± 1.6; DAB: 42.1 ± 1.3; ADA: 42.4 ± 1.4; BDA: 41.1 ± 1.4; BDB: 42.8 ± 1.6; ADB: 41.4 ± 1.4; DAD: 45.0 ± 1.2; BAD: 42.5 ± 1.4; DBD: 44.3 ± 1.3; ABD: 43.4 ± 1.4.

Behavioral and neurophysiological data were analyzed using mixed effects ANOVAs comprising the within-subject factors “experimental condition” (BI vs. BASE) and “electrode” (wherever applicable). “Group” (choice vs. switching vs. control group) was used as a between-subjects factor. Separate ANOVAs were calculated for each behavioral (RT and accuracy) and neurophysiological measure (ERPs). Greenhouse–Geisser correction was applied whenever necessary. Values are provided as means ± SDs. *Post hoc* tests were two-sided and Bonferroni-corrected, whenever necessary. All included variables were normally distributed as tested with Kolmogorov–Smirnov tests (all *z* < 0.9; *p* > 0.3).

## Results

We compared the behavioral and neurophysiological data obtained from an adapted BI paradigm (Koch et al., 2004) between three groups which had either undergone a choice-depletion, a switching-depletion, or no depletion prior to the experimental paradigm. The BI effect was assessed by comparing trial triplets with a task repetition of the n-2 trial (BI) to triplets lacking this repetition (BASE).

### Behavioral Data

**Figure 2:**
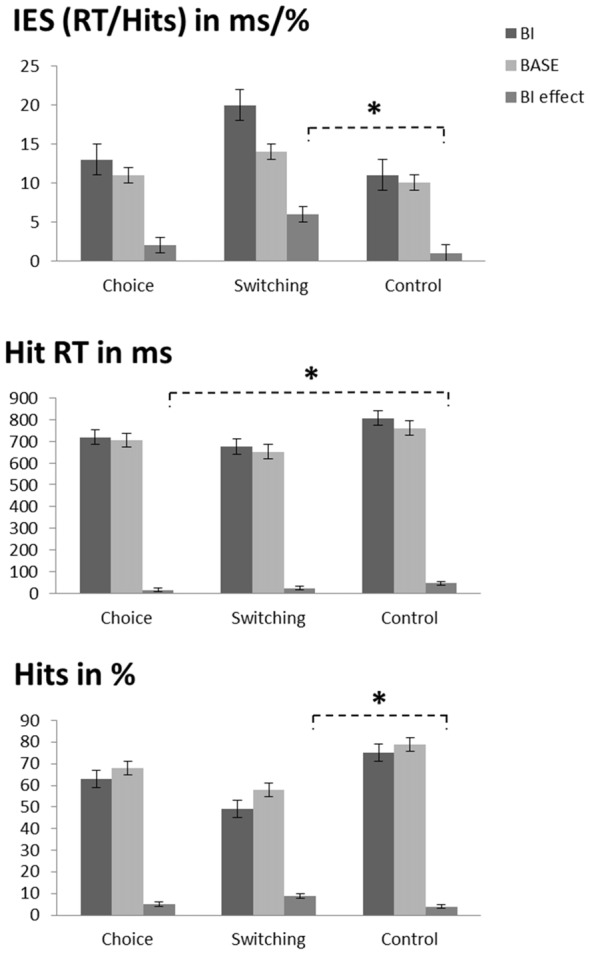
Illustration of the behavioral data. Top graph: inverse efficiency score (IES, which is the hit reaction time (RT) divided by the percentage of hits) as a function of condition (BI vs. BASE) and group (choice vs. switching vs. control). The asterisk denotes the interaction of condition and group (i.e., the larger BI effect in the switching group as compared to the control group). Middle graph: hit RTs as a function of condition (BI vs. BASE) and group (choice vs. switching vs. control). The asterisk denotes the interaction of condition and group (i.e., the smaller BI effect in the choice group as compared to the control group). Bottom graph: accuracy as a function of condition (BI vs. BASE) and group (choice vs. switching vs. control). The asterisk denotes the interaction of condition and group (i.e., the larger BI effect in the switching group as compared to the control group). Please note that only triplets with correct responses in all three trials were included in the analyses, thus decreasing the hit rates. As a consequence, the chance level is at 12.5% when assuming a 50% chance level for each individual trial of the analyzed triplets.

The repeated measures ANOVA on performance accuracy (percentage of hits for trial triplets; please note that the chance level is at 12.5%, not 50% in this context; see Table 1 and Figure 2) revealed a main effect of “experimental condition” (*F*_(1,72)_ = 58.84; *p* < 0.001; *η*^2^ = 0.450) showing that accuracy was higher in the BASE condition (68.2% ± 18.2) than in the BI condition (62.3% ± 21.6). A main effect of “group” (*F*_(2,72)_ = 11.52; *p* < 0.001; *η*^2^ = 0.242) showed that performance accuracy in the switching group (53.4% ± 20.2) was lower than the other two groups (*choice group*: 65.5% ± 19.4, *post hoc* comparisons: *p* = 0.047; *control group*: 76.9% ± 10.7, *post hoc* comparisons: *p* < 0.001). There was no difference between the choice group and the control group (*post hoc* comparisons: *p* = 0.069). Interestingly, an interaction of “experimental condition × group” (*F*_(2,72)_ = 3.85; *p* = 0.026; *η*^2^ = 0.097) showed that the BI effect (i.e., the experimental condition difference (Hits_BASE_ − Hits_BI_) was larger in the switching group (8.9% ± 7.2) than in the control group (3.9% ± 6.5; *post hoc* comparisons: *p* = 0.031). The intermediate BI effect of the choice group did not differ from the other two groups (*post hoc* comparisons: *compared to the switching group*: *p* = 0.122; *compared to the control group*: *p* > 0.900).

**Table 1:** Behavioral data.

	BI		BASE	
	RT (hits)	IES	RT (hits)	IES
Choice	720 ms ± 35 (63.0% ± 3.8)	13 ± 2	706 ms ± 33 (68.0% ± 3.3)	11 ± 1
Switching	676 ms ± 35 (48.9% ± 3.8)	20 ± 2	653 ms ± 33 (57.8% ± 3.3)	14 ± 1
Control	808 ms ± 35 (74.9% ± 3.8)	11 ± 2	762 ms ± 33 (78.8% ± 3.3)	10 ± 1

*Reaction time (RT), accuracy (percentage of hits) and IES (RT divided by the percentage of hits) as a function of condition (backward inhibition, BI vs. baseline, BASE) and group (choice vs. switching vs. control). Please note that only triplets with correct responses in all three trials were included in the analyses, thus decreasing the hit rates. Of note, the chance level would be at 12.5% when assuming a 50% chance level for each individual trial of the analyzed triplets*.

For the RTs (see Table 1 and Figure 2), the repeated measures ANOVA revealed a main effect of “experimental condition” (*F*_(1,72)_ = 33.82; *p* < 0.001; *η*^2^ = 0.320) showing that RTs were slower in the BI condition (734 ms ± 179) than in the BASE condition (706 ms ± 170). A main effect of “group” (*F*_(2,72)_ = 3.22; *p* = 0.046; *η*^2^ = 0.082) showed that RTs in the switching group (664 ms ± 88) were faster than in the control group (785 ms ± 227; *post hoc* comparisons: *p* = 0.041). There were no differences between the intermediate RTs of the choice group and the other two groups (*post hoc* comparisons: *compared to the switching group*: *p* = 0.931; *compared to the control group*: *p* = 0.412). An interaction of “experimental condition × group” (*F*_(2,72)_ = 3.82; *p* = 0.027; *η*^2^ = 0.096) revealed that the choice group (14 ms ± 29) had a smaller BI effect (RT_BI_ − RT_BASE_) than the control group (46 ms ± 43; *post hoc* comparisons: *p* = 0.027). There were no differences between the switching group and the other two groups in terms of the BI effect. (*post hoc* comparisons: *compared to the choice group*: *p* > 0.900; *compared to the control group*: *p* = 0.172).

To rule out that the observed effects were caused by a speed-accuracy tradeoff, we additionally investigated the speed-accuracy ratio (RT divided by the percentage of hits; separately calculated for each experimental condition), which is conducted as an inverse efficiency score (IES): the smaller this score, the more efficient the performance (see Table 1 and Figure 2). Matching the RT results, a main effect of “experimental condition” (*F*_(1,72)_ = 18.02; *p* < 0.001; *η*^2^ = 0.200) showed that the IES was larger (less efficient) in the BI condition (15 ± 12) than in the BASE condition (11 ± 6). Importantly, a main effect of “group” (*F*_(2,72)_ = 3.55; *p* = 0.034; *η*^2^ = 0.090) revealed that the IES in the switching group (16 ± 11) was larger (less efficient) than in the control group (10 ± 3; *p* = 0.030). No differences between the intermediate IES scores of the choice group and the other two groups were present (*post hoc* comparisons: all *p* > 0.240). Moreover, an interaction of “experimental condition × group” (*F*_(2,72)_ = 3.79; *p* = 0.027; *η*^2^ = 0.095) showed that the BI effect (IES_BI_ − IES_BASE_) was larger in the switching group (6 ± 10) than in the control group (1 ± 1; *post hoc* comparisons: *p* = 0.030). The BI effect in the choice group did not vary from the switching group (*post hoc* comparisons: *p* = 0.154) or the control group (*post hoc* comparisons: *p* > 0.900).

Taken together, the behavioral data are at odds with the ego depletion theory as we did not find the biggest impairments in the choice-depletion group. Instead, only the participants of the switching-depletion group responded less efficiently and had a larger BI effect than the non-depleted control group.

### Neurophysiological Data

To determine which neurophysiological mechanisms underlie the observed depletion effects, and how depletion modulates the BI effect, the amplitudes of P1, N1, P2, N2 and P3 ERPs were examined.

#### Early Attentional Processing of the Cue Stimulus

For the cue-elicited P1 at electrodes P7/P8, the mixed effects ANOVA revealed a main effect of “electrode” (*F*_(1,72)_ = 12.95; *p* = 0.001; *η*^2^ = 0.152). The cue P1 was larger at electrode P8 (28.40 μV/m^2^ ± 19.55) than at electrode P7 (21.46 μV/m^2^ ± 12.16). No other significant effects were present (all *F* < 3.10; all *p* > 0.051). For the cue-elicited N1 at electrodes P7/P8, no significant effects were found (all *F* < 3.85; all *p* > 0.054).

#### Early Attentional Processing of the Target Stimulus

Analyzing the target-elicited P1 at electrodes P7/P8 revealed no significant effects (all *F* < 2.13; all *p* > 0.127).

For the target-elicited N1 at electrodes P7/P8, a main effect of “experimental condition” (*F*_(1,72)_ = 6.37; *p* = 0.014; *η*^2^ = 0.081) showed that the N1 was more negative in the BI (−6.85 μV/m^2^ ± 16.68) than in the BASE condition (−4.84 μV/m^2^ ± 18.00). Aside from this, an interaction of “experimental condition × group × electrode” (*F*_(2,72)_ = 3.81; *p* = 0.027; *η*^2^ = 0.096) revealed that the N1 at electrode P7 was more negative in the BI than in the BASE condition in the switching group (BI: −15.88 μV/m^2^ ± 22.63; BASE: −11.20 μV/m^2^ ± 22.02; *t*_(24)_ = −2.18; *p* = 0.039) and the control group (BI: −9.60 μV/m^2^ ± 21.23; BASE: −6.31 μV/m^2^ ± 23.09; *t*_(24)_ = −2.06; *p* = 0.050), but not in the choice group (BI: −4.69 μV/m^2^ ± 18.61; BASE: −4.78 μV/m^2^ ± 17.29; *t* = 0.06; *p* = 0.954). No such effects were found for electrode P8 (all *t* < 1.91; all *p* > 0.068).

**Figure 3:**
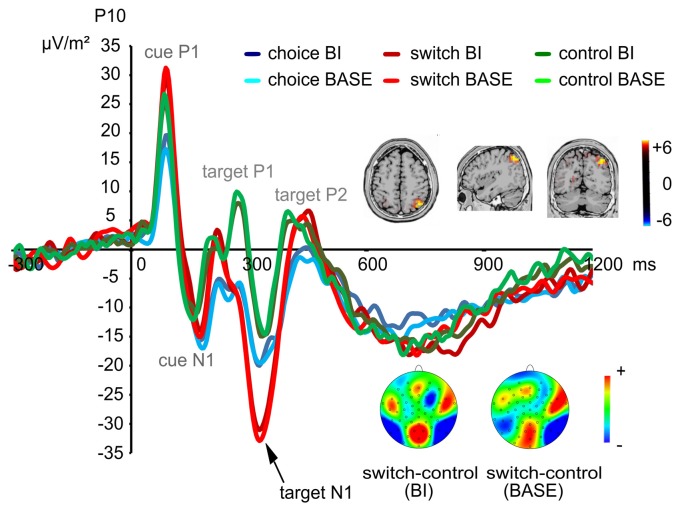
Early visual attentional event-related potentials (ERPs) evoked by the target stimuli at electrode P10. Time point zero denotes the onset of the cue; the target stimulus was added to the visual array 100 ms later. Hence, the first two peaks show the P1 and N1 elicited by the cue while the following three peaks show the P1, N1 and P2 elicited by the target. As shown, a significant group difference (switching > control group) was found for the target N1. This difference was rooted in activity changes in the precuneus and the superior parietal cortex (BA7).

Based on the activities shown in the scalp topography, we also analyzed the target N1 at electrodes P9/P10. A main effect of “group” (*F*_(2,72)_ = 4.02; *p* = 0.022; *η*^2^ = 0.100) showed that the target N1 was more negative in the switching group (−29.52 μV/m^2^ ± 22.35) than in the control group (−14.62 μV/m^2^ ± 17.95; *post hoc* comparisons: *p* = 0.021). When comparing the choice group with the other two groups, no group differences could be found (*post hoc* comparisons: all *p* > 0.182). An interaction effect of “electrode × group” (*F*_(2,72)_ = 3.29; *p* = 0.043; *η*^2^ = 0.084) revealed that the group difference between the switching group (−30.71 μV/m^2^ ± 21.23) and the control group (−9.72 μV/m^2^ ± 17.42) was only found at electrode P10 (*F*_(2,72)_ = 7.07; *p* = 0.002; *η*^2^ = 0.164, *post hoc* comparisons: *p* = 0.001). The target N1 in the choice group was comparable to the other two groups at electrode P10 (*post hoc* comparisons: all *p* > 0.112; The target N1 at electrode P10 is shown in Figure 3). No group differences were found at electrode P9 (*F*_(2,72)_ = 1.31; *p* = 0.275; *η*^2^ = 0.035). Moreover, an interaction effect of “experimental condition × group × electrode” (*F*_(2,72)_ = 8.95; *p* < 0.001; *η*^2^ = 0.199) was found. In the BI condition, the target N1 was more negative in the switching group (−29.98 μV/m^2^ ± 21.83) than in the control group (−14.70 μV/m^2^ ± 16.04) at both electrodes (*F*_(2,72)_ = 4.70; *p* = 0.012; *η*^2^ = 0.115, *post hoc* comparisons: *p* = 0.013). However, this difference could only be found at electrode P10 in the BASE condition (*F*_(2,72)_ = 6.96; *p* = 0.002; *η*^2^ = 0.162, *post hoc* comparisons: *p* = 0.001, *switching group*: −31.51 μV/m^2^ ± 22.30, *control group*: −9.09 μV/m^2^ ± 19.69). No differences between the choice group and the other two groups were found (*post hoc* comparisons: all *p* > 0.099). No other significant main effects or interactions were found on the target N1 (all *F* < 2.69; all *p* > 0.107). The sLORETA analysis revealed that this difference between the switching group and the control group was due to activity changes in the precuneus and the superior parietal cortex (BA7).

For the target-elicited P2 at electrodes P7/P8, an interaction of “experimental condition × electrode” (*F*_(1,72)_ = 4.44; *p* = 0.039; *η*^2^ = 0.058) showed that the target P2 in the BASE condition (12.44 μV/m^2^ ± 18.00) was larger than in the BI condition (10.47 μV/m^2^ ± 18.06) at electrode P7 (*t*_(74)_ = −2.50; *p* = 0.015). No other significant effects were found (all *F* < 1.97; all *p* > 0.165).

#### Conflict Processing

For the cue-elicited N2 at electrode Cz, no significant effects were found (all *F* < 2.83; all *p* > 0.066). For the target-elicited N2 at electrode Cz, a main effect of “experimental condition” (*F*_(1,72)_ = 6.42; *p* = 0.013; *η*^2^ = 0.082) showed that the target N2 in the BASE condition (−15.71 μV/m^2^ ± 12.02) was more negative than in the BI condition (−13.41 μV/m^2^ ± 14.40). No other significant effects were revealed (all *F* < 0.91; all *p* > 0.406). To rule out that the observed effect on the target N2 was affected by the temporally overlapping target P2, the correlation between the two ERPs was calculated. This however yielded no significant correlation (*r* = −0.066, *p* = 0.574).

#### Stimulus Evaluation, Response Selection and Context Updating

**Figure 4:**
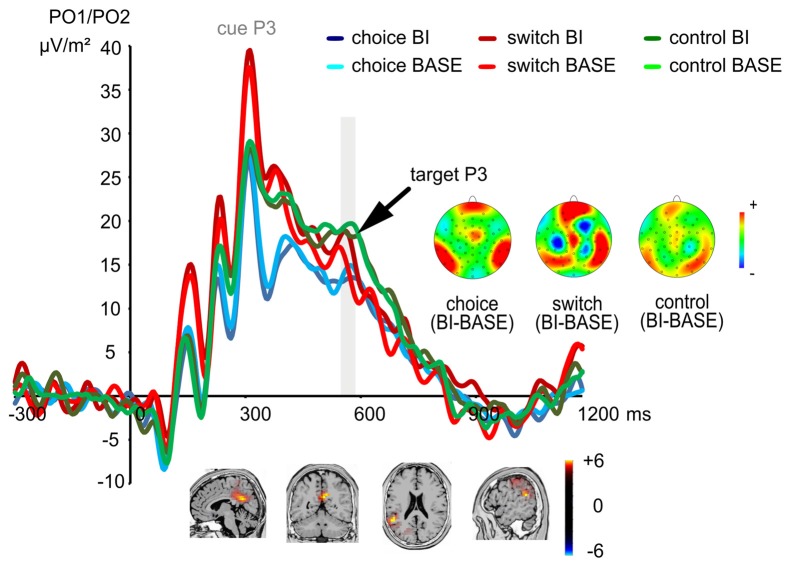
The P3 ERP at electrodes PO1 and PO2. The cue-elicited P3 did not show any significant effects, while the target-elicited P3 showed a significant condition difference (BI vs. BASE) only in the switching group, but not in other two groups. The time interval used for quantification of the target-elicited P3 is denoted in gray color. This group difference between the switching group and the other two groups in terms of the magnitude of the BI effect was related to activity modulations in the dorsal posterior cingulate area (BA31) and the right inferior parietal lobe (BA40).

The P3 ERP is shown in Figure 4.

Analyzing the P3 associated with the cue stimulus, a main effect of “electrode” (*F*_(1,72)_ = 4.69; *p* = 0.034; *η*^2^ = 0.061) was found showing that the cue P3 was larger at electrode PO2 (31.41 μV/m^2^ ± 19.96) than at electrode PO1 (28.16 μV/m^2^ ± 20.88). No other significant effects were found (all *F* < 2.84; all *p* > 0.065).

For the P3 associated with the target stimulus, a main effect of “electrode” (*F*_(1,72)_ = 7.01; *p* = 0.010; *η*^2^ = 0.089) showed that the P3 was larger at electrode PO1 (19.24 μV/m^2^ ± 15.10) than at electrode PO2 (16.50 μV/m^2^ ± 11.79). Importantly, an interaction of “experimental condition × group” (*F*_(2,72)_ = 4.08; *p* = 0.021; *η*^2^ = 0.102) showed that an experimental condition difference was only found in the switching group (*switching group*: *t*_(24)_ = 2.30; *p* = 0.030), but not in the other two groups (*choice group*: *t*_(24)_ = −0.68; *p* = 0.502; *control group*: *t*_(24)_ = −1.60; *p* = 0.124). The target P3 of the switching group was larger in the BI condition (19.38 μV/m^2^ ± 16.41) as compared to the BASE condition (16.22 μV/m^2^ ± 15.94). The sLORETA analysis showed that this group difference between the switching group and the other two groups in terms of the magnitude of the target P3 BI effect resulted from activity modulations in the dorsal posterior cingulate area (BA31) and the right inferior parietal lobe (BA40). No other effects were significant (all *F* < 1.55; all *p* > 0.217).

## Discussion

In the current study, we investigated whether making self-referred choices leads to domain-unspecific (ego) depletion that impairs various self-regulation activities even in unrelated cognitive domains. As inhibitory processes are essential to self-regulation and sensitive to self-regulatory depletion, we put a focus on how depletion modulates inhibitory control in a task switching context.

As explained in the “Introduction” section, the resource model/ego depletion theory would suggest that the choice group should have shown the worst performance/largest performance impairment as compared to the other two groups, because decision-making is costly and consumes the self-regulatory resource, which should have left the participants in the choice group less able to regulate themselves. Yet, we did not find a choice-induced depletion effect. A potential explanation for this could be that the pleasantness induced by choosing between attractive stimuli might eliminate the impact of choosing (Moller et al., 2006; Vohs et al., 2008). However, pleasant choices are also depleting when participants have made choices for a relatively long time, i.e., at least 12 min (Vohs et al., 2008). Since the choice task in the current study took about 20 min, the absence of depletion in the choice group cannot be explained by the pleasantness induced by choices alone. In contrast to this, we found the switching group to respond more impulsively and less efficiently in the BI paradigm than the other two groups (which did not differ). The fact that the switching group, but not the choice group, differed from the non-depleted controls supports the assumption of a domain-specific depletion because both the switching-based depletion task and the experimental BI paradigm assess the same cognitive domain (i.e., cognitive flexibility/task switching). In other words, the switching-based depletion task and the BI task (our measure of self-regulation) both require flexible task switching and thus share similar cognitive processes. It is therefore likely that conducting the switching task depletes an important cognitive resource needed for task switching. As a consequence, the subsequent performance on the BI task should be impaired as it also requires flexible task switching. Based on this notion, we suppose that the depletion effect is at least partly domain-specific and can thus mainly be observed when the depleting task and the subsequent task rely on the same cognitive processes. This is also in line with findings by Tuk et al. (2015), who demonstrated depletion effects in case of sequentially carried out control tasks requiring inhibition, which is known to also play an important role for set shifting/task switching (Diamond, 2013). Other than what is suggested by the ego depletion theory, choice-making alone does not seem to be sufficient to deplete a general cognitive resource to a degree where this produces detrimental effects in an unrelated cognitive control domain. Also, we did not find any improvement of self-regulation as reported by Dewitte et al. (2009) or Converse and DeShon (2009), who assumed that a gradual adaptation process may yield improved self-regulation after repeatedly exerting it. One might try to argue that we did not find such as effect because we did not provide our participants with enough trials/time to adapt. Yet, we used decisively more depletion trials than most of these studies, so that it seems more likely that findings from consumer research do not directly translate to basic cognitive control functions, especially when those are not paired with inherent rewards (as the products used in consumer research often are). As we examined the ego depletion effect by using two subsequent control tasks we also did not find any beneficial spill-over effects, which may only be observed in some cognitive control domains, such as inhibition, any have only been observed when self-control is simultaneously exerted in multiple domains (Tuk et al., 2015).

Importantly, the neurophysiological data parallels our behavioral findings and shows which cognitive sub-processes are modulated: The target-evoked N1 amplitudes at parietal sites were larger in the switching group than in the control group, but there were no significant differences between the choice group and the control group. The N1 has been suggested to be strongly modulated by attentional selection processes such as focusing of attention towards task-relevant stimuli (Luck et al., 1990; Hillyard and Anllo-Vento, 1998; Herrmann and Knight, 2001; Beste et al., 2010a; Schneider et al., 2012). In addition, source localization analyses revealed that the differences observed in the switching group resulted from activation differences in precuneus and superior parietal lobe (BA7), which plays a crucial role in visual selection (Giesbrecht et al., 2003; Chambers et al., 2006; Oh and Leung, 2010). It may therefore be assumed that the participants in the switching group had problems in keeping their attention focused on the current task and its relevant information due to a switching-based depletion effect. In contrast, participants in the choice group did not show any impairment in selective attention.

The finding of a larger BI effect/experimental condition difference in the switching group was also paralleled in the amplitudes of the target P3. The P3 component has been assumed to reflect processes between stimulus evaluation and responding; i.e., the response selection process (Verleger et al., 2005; Twomey et al., 2015). Since the recently abandoned task becomes relevant again in the BI condition, it could be more difficult for the depleted participants in the switching group to choose an appropriate stimulus-response mapping in the BI condition, thus resulting in a larger P3 in the BI condition than in the BASE condition. In the context of task switching, the P3 component has also been proposed to be related to context updating, organization and implementation of the new task-set (Polich, 2007; Gajewski et al., 2010, 2011; Gajewski and Falkenstein, 2011; Wolff et al., 2016). When a recently abandoned task becomes relevant again in the BI condition, the switching-depleted participants seem more likely to fail to re-evaluate the stimuli and to update the context, which should have led to a larger P3 in the BI condition than the BASE condition. The group differences in the BI modulation of the P3 component are due to activity changes in the dorsal posterior cingulate area (BA31) and right inferior parietal lobe (BA40) including the temporo-parietal junction (TPJ). The dorsal posterior cingulate area serves evaluation and updating (Seo and Lee, 2007; Chen et al., 2010; Stern et al., 2010; Mende-Siedlecki and Todorov, 2016) while uncertainty during updating is associated with increased activity in the dorsal anterior cingulate cortex (Stern et al., 2010; Mende-Siedlecki and Todorov, 2016). In the current study, the different activities in this area may reflect depletion-related uncertainty and difficulty in re-evaluating the stimuli or selecting responses. The TPJ has also been reported to be involved in attentional control for contextual updating (Pessoa et al., 2009; Karch et al., 2010; Geng and Vossel, 2013; Dippel et al., 2016) and is related to modulations in the P3 component (Verleger et al., 1994; Karch et al., 2010; Mückschel et al., 2014). The TPJ may mediate the updating of an internal model which initiates context and task-appropriate actions (Geng and Vossel, 2013). This matches our results well by showing that the observed depletion in the switching group seems to affect attentional processes, which are necessary to update the environmental context (as in the BI condition). Our finding that self-referred choice-making is not generally detrimental to one’s subsequent self-regulation counters the theoretical assumption that the same resource is used for many diverse self-regulation activities. Instead, the observed depletion in the switching group provides evidence in favor of a domain-specific depletion model. Based thereon, we suspect that there are separate and independent resource for various cognitive processes rather than a joint resource for all self-regulation activities. Our domain-specific depletion model provides a possible explanation for the findings of Carter et al. (2015) who reported that only certain behaviors rather than any act of self-control show the depletion effect. As studies replicating depletion effect have often focused only on a single combination of depleting and dependent task and used the same standardized tests such as food consumption rather than applying other more classic self-regulation tasks (Carter et al., 2015), our finding encourage further studies to use different self-regulation tasks and multiple combinations of depleting and dependent tasks to examine depletion effects.

Replicating previous data (Sinai et al., 2007; Zhang et al., 2016a,b), the target-elicited N1 amplitude was larger in the BI than the BASE condition, which may be attributed to increased attentional requirements to re-activate the recently abandoned task in the BI condition. The smaller P2 in the BI condition may be caused by the higher task demands in the BI condition requiring more attention than the BASE condition (García-Larrea et al., 1992; Crowley and Colrain, 2004; Sugimoto and Katayama, 2013). The smaller N2 in the BI condition could result from a larger switching-induced conflict on the previous trial as compared to the BASE condition (Gratton et al., 1992; Zhang et al., 2016a).

In summary, we were able to challenge the ego depletion theory in its current form. Opposing its predictions, we found stronger behavioral performance impairments following a domain-specific depletion than a depletion based on self-referred choices. This means that even though there might be a general component to self-regulatory behavior, there are also domain-specific resource contingents which are most strongly depleted by activities within this specific domain. This suggests at least partly separate and independent resources for various cognitive control processes rather than just one joint resource for all self-regulation activities.

## Author Contributions

RZ, A-KS and CB designed the study. RZ, AR and A-KS collected and analyzed the data. All authors contributed to the writing of the manuscript.

## Conflict of Interest Statement

The authors declare that the research was conducted in the absence of any commercial or financial relationships that could be construed as a potential conflict of interest.

## Footnotes

^1^Importantly, inhibition is the cognitive faculty underlying all kinds of self-regulation, including things like refraining from eating cookies, which has previously often been used as a measure of self-control.
